# Risk factors for nonresponsive hydration in patients with spinal cerebrospinal fluid leakage

**DOI:** 10.1186/s12883-021-02464-6

**Published:** 2021-11-03

**Authors:** Hung-Chieh Chen, Po-Lin Chen, Jyh-Wen Chai, Chieh-Lin Jerry Teng

**Affiliations:** 1 Department of Radiology, Taichung Veterans General Hospital, Taiwan, 1650 Taiwan Boulevard Sect. 4, Taichung, Taiwan 407; 2 School of Medicine, National Yang-Ming Chiao-Tung University, Taipei, Taiwan; 3 Department of Neurology, Taichung Veterans General Hospital, Taiwan, Taichung, Taiwan; 4 College of Medicine, China Medical University, Taichung, Taiwan; 5 Division of Hematology/Medial Oncology, Department of Medicine, Taichung Veterans General Hospital, Taiwan, 1650 Taiwan Boulevard Sect. 4, Taichung, Taiwan 407; 6 School of Medicine, Chung Shan Medical University, Taichung, Taiwan; 7 College of Medicine, National Chung Hsing University, Taichung, Taiwan

**Keywords:** Cerebrospinal fluid, Leakage, Epidural blood patch, Hydration

## Abstract

**Background:**

Spinal cerebrospinal fluid (CSF) leakage is frequently encountered clinically after lumbar puncture or spontaneous events. Although some patients recover without treatment or after intensive hydration, some require an epidural blood patch (EBP). The risks of nonresponsive hydration remain unknown. Therefore, we identified the risk factors for patients with spinal CSF leakage nonresponsive to hydration.

**Methods:**

We retrospectively reviewed patients diagnosed with spinal CSF leakage between January 2010 and March 2021. Clinical data, including patient age, sex, etiology, and radiological indications in magnetic resonance imaging, were compared between patients who were responsive and non-responsive to hydration.

**Results:**

Of the 74 patients with spinal CSF leakage, 25 were responsive to hydration and 49 required EBP. Patients who were nonresponsive to hydration were older (39.27 vs. 34.32 years, *P* = 0.01), had a higher percentage of spontaneous intracranial hypotension (93.88% vs. 68.00%, *P* = 0.005), had more spinal CSF leakage (12.04 vs. 8.04, *P* = 0.01), and had a higher percentage of dural sinus engorgement (81.63% vs. 60.00%, *P* = 0.044). Spontaneous intracranial hypotension (odds ratio [OR]: 4.63; 95% confidence interval [CI]: 1.00-21.38) and having ≥9 spinal CSF leakages (OR: 3.29; 95% CI: 1.08-10.01), as indicated by magnetic resonance myelography, are considered risk factors for noneffective hydration.

**Conclusions:**

Patients with spinal CSF leakage who have spontaneous intracranial hypotension and those with ≥9 spinal CSF leakages are considered at risk for noneffective hydration. EBP should be considered early in these patients.

## Background

Spinal cerebrospinal fluid (CSF) leakage is usually encountered in clinical settings. The major etiologies include post-dural puncture headache (PDPH) or a spontaneous event [1, 2], with incidences of approximately 12–40% [3] and 0.05% [4], respectively.

The possibility of spinal CSF leakage must be carefully excluded in patients with orthostatic headaches. A definitive diagnosis relies on standard radiological findings; definitive CSF leakage can be determined using spinal magnetic resonance myelography (MRM) [5–8] to indicate abnormal spinal CSF signals along spinal neural sleeves or the accumulation of abnormal CSF in the epidural space. Diffused pachymeningeal enhancement [9, 10], pituitary hyperemia [11], dural sinus engorgement [9, 10], brain descent [10], subdural effusion, and subdural hematoma (SDH) [12] are common radiological findings on brain magnetic resonance imaging (MRI) in patients with CSF leakage. Patients may also have abnormal fluid accumulation at the C1–2 junction [13] and epidural venous plexus engorgement on a spinal MRI [10].

Although patients can spontaneously recover after bed rest, SDH with brain herniation or sinus thrombosis [14, 15] are severe complications if the disease is not recognized and treated. Intensive intravenous hydration is the standard treatment for symptomatic spinal CSF leakage [16]. Patients who are nonresponsive to hydration require an epidural blood patch (EBP) [1, 17]. Evaluation of treatment response relies on the improvement of clinical symptoms. However, the risk factors for patients who are nonresponsive to hydration and require further EBP are unclear. These patients usually experience longer hospitalizations and may have a higher risk of complications.

In this study, we identified the risk factors for patients with spinal CSF leakage who were nonresponsive to hydration.

## Methods

### Participants

**Fig. 1:**
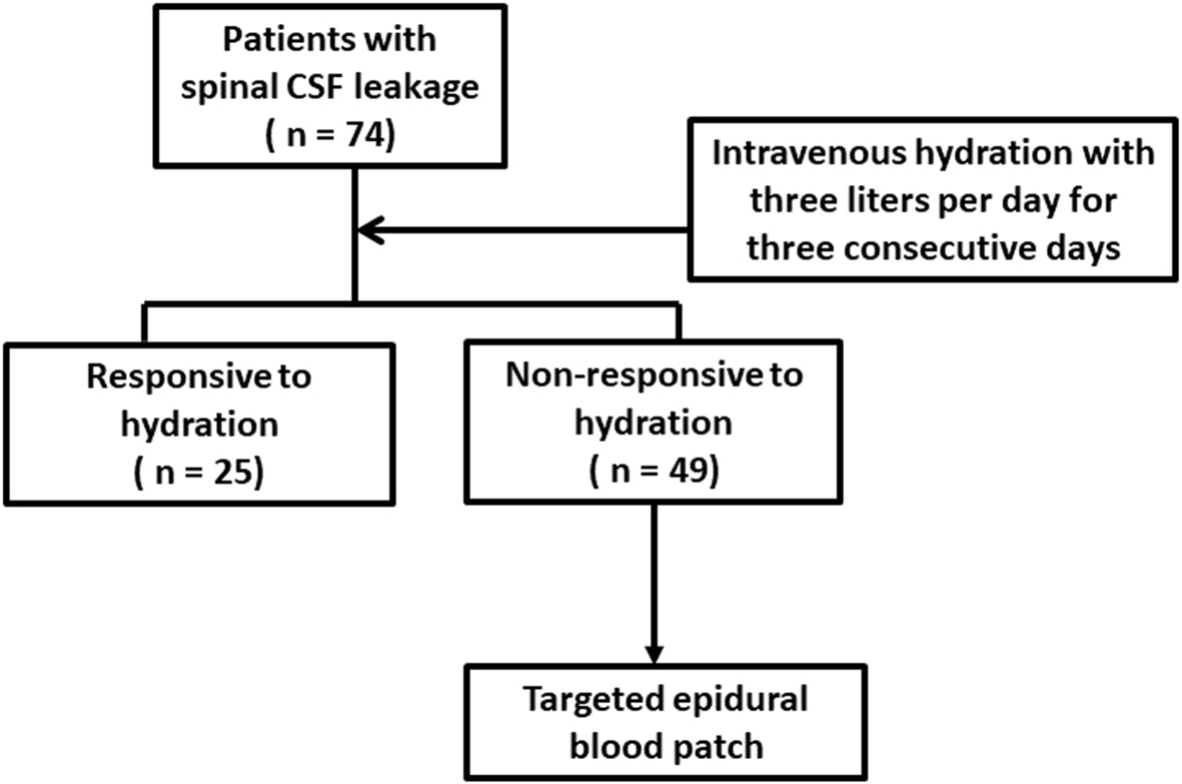
The algorithm for management in this study

This study was conducted in accordance with the principles of the Declaration of Helsinki. The Institutional Review Board of Taichung Veterans General Hospital approved this study (CE21140B). Due to the retrospective design, the need for informed consent was waived by the Institutional Review Board of Taichung Veterans General Hospital**.** Patients who were referred for MRI studies due to orthostatic headache and were diagnosed with spinal CSF leakage between January 2010 and March 2021 were recruited to our study. Their etiologies include PDPH and spontaneous intracranial hypotension (SIH). During hospitalization, the patients received intravenous fluid hydration at 3 L per day for three consecutive days. Targeted EBP was administered if the patients were nonresponsive to hydration (Fig. 1).

The etiology, age at symptom onset, sex, headache score measurement using the visual analogue scale upon presentation, duration between symptom onset and MRI examination (onset–diagnosis interval), and hospitalization duration were collected from medical records for analysis.

### Neuroimaging

A 1.5-T MRI scanner (MAGNETOM Aera, Siemens Healthcare, Erlangen, Germany) was used. All patients received whole-spine MRM and spine and conventional brain MRI simultaneously upon presentation. Conventional brain MRI included axial spin-echo T1WI (repetition time (TR)/ echo time (TE), 500/10), axial fast spin-echo T2-weighted images (TR/TE 3200/115), and Gd-enhanced spin-echo T1WI images in the axial, sagittal, and coronal planes. Three-dimensional volumetric interpolated breath-hold examination and Gd-enhanced T1WI focusing on C-T spine level were also obtained to determine if spinal epidural venous engorgement was present. Whole-spine MRM was performed with 3-dimensional sampling perfection and optimized contrast using variable flip-angle evolution (3D-SPACE) sequences. The MRM parameters were as follows: TR = 3000 ms, TE = 560 ms, isotropic voxel size = 0.9 mm^3^, matrix size = 320 × 320 px, and field of view = 200 mm. Fat suppression was employed, and generalized auto-calibrating partially parallel acquisition imaging reconstruction with an acceleration factor of 2 was used. Images were acquired volumetrically in the coronal plane of the cervical-to-thoracic and thoracic-to-lumbar regions of the spine parallel to the spinal curve with some overlapping.

Qualitative brain MRI parameters were evaluated. Pituitary hyperemia was defined as the convex shape of the pituitary gland. Venous engorgement appeared as a convex upper surface of the sinus confluence on the midline of the sagittal view of post-contrast T1WI. Diffuse pachymeningeal enhancement was defined as the presence of continually enhanced dura matter. The presence or absence of SDH was also recorded.

The number of abnormal spinal CSF signals along each spinal neural sleeve on MRM was recorded as the number of spinal CSF leakages. For example, if abnormal spinal CSF signals were present on MRM at both C2–3 and C3–4, the number of CSF leakages was four. Spinal epidural fluid accumulation was defined by the number of vertebral bodies where the abnormal epidural fluid was on MRM. All imaging findings were interpreted by an experienced neuroradiologist (H.C. Chen).

### Treatment response evaluation

Patients were classified into two groups based on response to treatment. Patients who could not tolerate their clinical symptoms after hydration and who underwent target EBP were defined as having noneffective hydration. Patients who could tolerate their clinical symptoms and did not have symptom recurrence within 1 month were defined as having hydration effectiveness.

### Statistical analyses

All data were analyzed using SPSS software (v21; SPSS, Chicago, IL, USA). Demographic data and MRI indications were compared between the hydration-effective and hydration-noneffective groups. Kolmogorov–Smirnov tests were used to verify whether the continuous variables were normally distributed. Age, headache scores, onset-diagnosis interval, number of spinal CSF leakages, and levels of spinal epidural fluid accumulation were not normally distributed and were, therefore, analyzed with nonparametric Mann–Whitney tests. Fisher’s exact test and the chi-square test were used for nominal variables. We used the receiver operating characteristic (ROC) curve to calculate the threshold for the number of CSF leakages. The risk factors for noneffective hydration were analyzed using univariate logistic regression, quantified as odds ratios (ORs) and their corresponding 95% confidence intervals (CIs). Those factors with *P* < 0.05 were included in a multivariable logistic regression using the forward method. All tests were two-tailed, with statistical significance defined at *P* < 0.05.

## Results

### Clinical profile

From January 2010 to March 2021, 74 patients were diagnosed using MRM due to at least one spinal CSF leak. At total of 25 patients (33.78%) recovered after hydration without further EBP. The other 49 patients (66.22%) could not tolerate their symptoms and underwent EBP treatment.

No considerable difference was observed with respect to sex, headache score, and onset–diagnosis interval between the hydration-effective and hydration-noneffective groups. Compared with the hydration-noneffective groups, the hydration-effective groups were younger and had a higher percentage of PDPH relative to SIH (Table 1).

**Table 1:** Demographic data of patients with spinal CSF leakage for hydration effectiveness

	Total (***n*** = 74)	Hydration effective (***n*** = 25)	Hydration non-effective (***n*** = 49)	***P***-value
**Age, years**^a^	37.59 ± 11.51	34.32 ± 14.49	39.27 ± 9.38	0.010
**Gender, n (%)**^b^				
Male	25 (33.78%)	6 (24.00%)	19 (38.78%)	0.204
Female	49 (66.22%)	19 (76.00%)	30 (61.22%)	
**Etiology, n (%)**^c^				
PDPH	11 (14.86%)	8 (32.00%)	3 (6.12%)	0.005
SIH	63 (85.14%)	17 (68.00%)	46 (93.88%)	
**Pain score**^a^	7.13 ± 1.85	6.86 ± 1.81	7.27 ± 1.87	0.408
**Onset-diagnosis Interval, days**^a^	20.20 ± 46.84	26.08 ± 71.49	17.20 ± 27.47	0.876
**Admission days**^a^	13.85 ± 8.63	10.16 ± 8.02	15.74 ± 8.39	0.000

Mann-Whitney test^a^. Chi-Square test^b^. Fisher’s Exact test^c^

Numeric data are presented as mean ± standard deviation

*PDPH* Post-dural puncture headache, *SIH* Spontaneous intracranial hypotension

### Neuroimaging

**Fig. 2:**
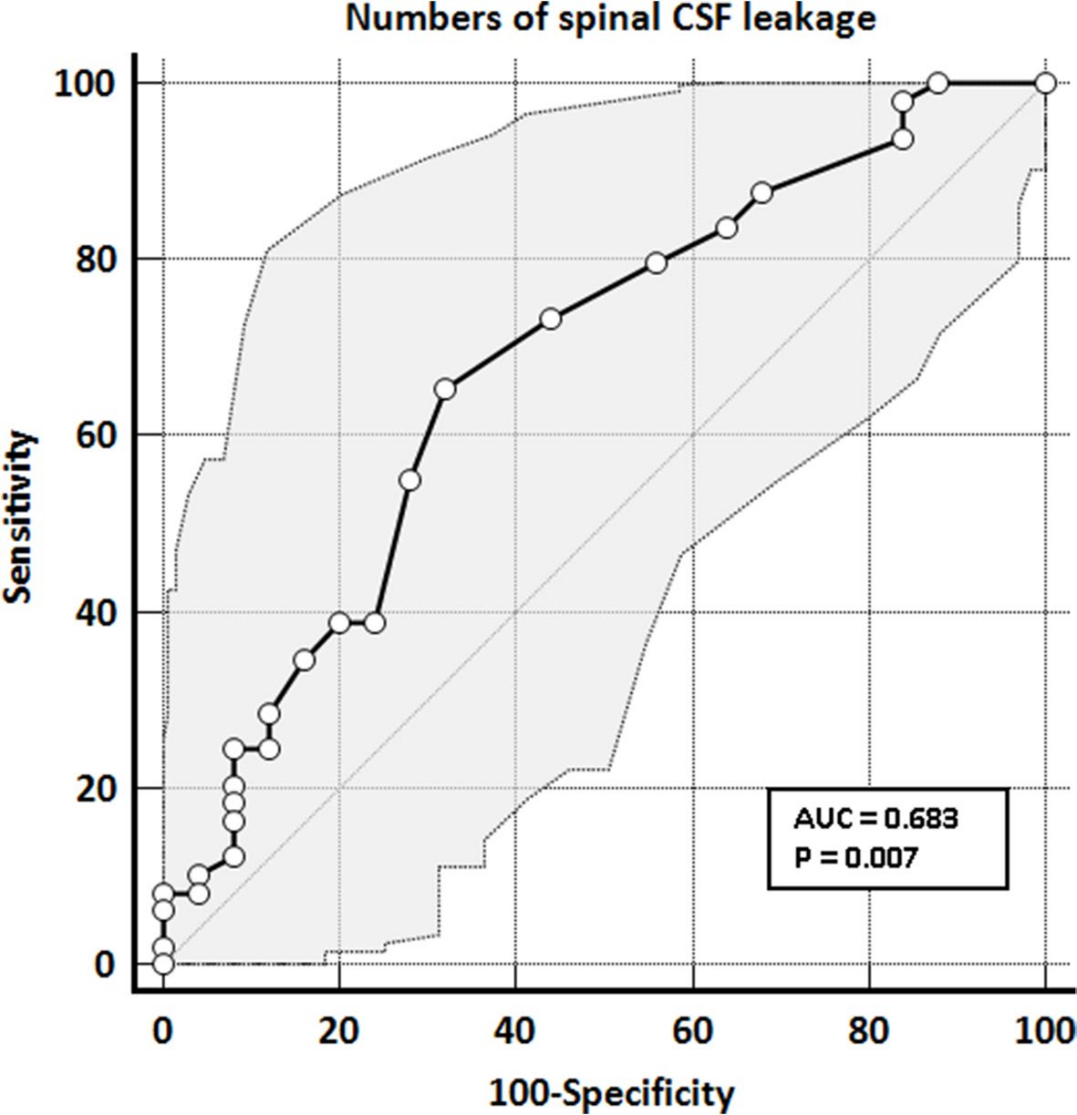
ROC curve for spinal CSF volume. Numbers for spinal CSF leakage for hydration non-effectiveness

Substantially greater numbers of spinal CSF leakage in the MRM and percentage of dural sinus engorgement were found in the hydration-noneffective group (Table 2). The cutoff value for the number of spinal CSF leakages, using ROC curve analysis, for prediction of noneffective hydration was nine (Fig. 2).

**Table 2:** Comparison of Clinical and MRI parameters for hydration effectiveness

	Total (n = 74)	hydration effective (n = 25)	hydration non-effective (n = 49)	***P***-value
**Numbers of spinal CSF leakage**^a^	10.69 ± 7.02	8.04 ± 5.95	12.04 ± 7.19	0.010
**Levels of spinal epidural fluid accumulation**^a^	6.76 ± 4.98	6.44 ± 4.92	6.92 ± 5.05	0.863
**Spine epidural venous engorgement, n (%)**^b^				0.563
No	35 (47.30%)	13 (52.00%)	22 (44.90%)	
Yes	39 (52.70%)	12 (48.00%)	27 (55.10%)	
**DPE, n (%)**^b^				0.167
No	22 (29.73%)	10 (40.00%)	12 (24.49%)	
Yes	52 (70.27%)	15 (60.00%)	37 (75.51%)	
**Brain descend, n (%)**^b^				0.191
No	52 (70.27%)	20 (80.00%)	32 (65.31%)	
Yes	22 (29.73%)	5 (20.00%)	17 (34.69%)	
**Dural sinus engorgement, n (%)**^b^				0.044
No	19 (25.68%)	10 (40.00%)	9 (18.37%)	
Yes	45 (60.81%)	15 (60.00%)	40 (81.63%)	
**SDH, n (%)**^c^				0.358
No	60 (81.08%)	22 (88.00%)	38 (77.55%)	
Yes	14 (18.92%)	3 (12.00%)	11 (22.45%)	
**Pituitary hyperemia, n (%)**^b^				0.570
No	33 (44.59%)	10 (40.00%)	23 (46.94%)	
Yes	41 (55.41%)	15 (60.00%)	26 (53.06%)	

Mann-Whitney test^a^. Chi-Square test^b^. Fisher’s Exact test^c^; Numerical data are presented as mean ± standard deviation

*DPE* Diffuse pachymeningeal enhancement, *SDH* Subdural hematoma

The multivariable analysis results for the risk factors for hydration non-effectiveness are summarized in Table 3. Patients who had SIH and those with ≥9 spinal CSF leakages were considered to have higher possibility of noneffective hydration; their ORs were 4.63 (95% CI 1.00-21.38)) and 3.29 (95% CI 1.08-10.01), respectively.

**Table 3:** Risk factors for noneffective hydration

	Univariate analysis			Multivariate analysis		
	OR	95% CI	***P***-value	OR	95% CI	***P***-value
**Age, years**	1.04	0.99–1.10	0.085			
**Etiology (SIH vs. PDPH)**	7.22	1.71–30.42	0.007	4.63	1.00–21.38	0.049
**Dural sinus engorgement (Yes vs. No)**	2.96	1.01–8.71	0.048			
**Numbers of spinal CSF leakage level (≥ 9 vs. < 9)**	4.17	1.43–12.12	0.009	3.29	1.08–10.01	0.036

*SIH* Spontaneous intracranial hypotension, *PDPH* Post-dural puncture headache, *OR* Odds ratio, *CI* Confidence interval

Because SIH is a risk factor for noneffective hydration, we further evaluated the parameters in patients with SIH (Table 4). No substantial difference was indicated for the clinical parameters of age, headache score, or onset-diagnosis interval between the hydration-effective and hydration-noneffective groups. The presence of radiological abnormalities, as indicated by the number of spinal CSF leakages, levels of spinal epidural fluid accumulation, spinal epidural venous engorgement, diffuse pachymeningeal enhancement, brain descent, dural sinus engorgement, SDH, and pituitary hyperemia, did not substantially differ between the hydration-effective and hydration-noneffective groups.

**Table 4:** Comparison of clinical and MRI parameters for hydration effectiveness in patients with SIH

	Total (***n*** = 63)	hydration effective (***n*** = 17)	hydration non-effective (***n*** = 46)	***P***-value
**Age, years**^a^	39.08 ± 11.25	37.94 ± 15.41	39.50 ± 9.45	0.135
**Headache score**^a^	7.16 ± 1.84	7.15 ± 1.82	7.16 ± 1.87	0.988
**Onset-diagnosis Interval, days**^a^	21.44 ± 50.32	31.18 ± 86.21	17.85 ± 28.23	0.969
**Numbers of spinal CSF leakage**^a^	11.41 ± 7.27	9.35 ± 6.72	12.17 ± 7.39	0.185
**Levels of spinal epidural fluid accumulation**^a^	7.08 ± 5.15	7.18 ± 5.42	7.04 ± 5.10	0.756
**Spine epidural venous engorgement, n (%)**^b^				0.921
No	29 (46.03%)	8 (47.06%)	21 (45.65%)	
Yes	34 (53.97%)	9 (52.94%)	25 (54.35%)	
**DPE, n (%)**^c^				0.523
No	15 (23.81%)	5 (29.41%)	10 (21.74%)	
Yes	48 (76.19%)	12 (70.59%)	36 (78.26%)	
**Brain descend, n (%)**^b^				0.577
No	41 (65.08%)	12 (70.59%)	29 (63.04%)	
Yes	22 (34.92%)	5 (29.41%)	17 (36.96%)	
**Dural sinus engorgement, n (%)**^c^				0.204
No	15 (23.81%)	6 (35.29%)	9 (19.57%)	
Yes	48 (76.19%)	11 (64.71%)	37 (80.43%)	
**SDH, n (%)**^c^				0.485
No	50 (79.37%)	15 (88.24%)	35 (76.19%)	
Yes	13 (20.63%)	2 (11.76%)	11 (23.91%)	
**Pituitary hyperemia, n (%)**^b^				0.299
No	29 (46.03%)	6 (35.29%)	23 (50.00%)	
Yes	34 (53.97%)	11 (64.71%)	23 (50.00%)	

Mann-Whitney test^a^. Chi-Square test^b^. Fisher’s Exact test^c^; numerical data are presented as mean ± standard deviation

*DPE* Diffuse pachymeningeal enhancement, *SDH* Subdural hematoma

### Case presentation

**Fig. 3:**
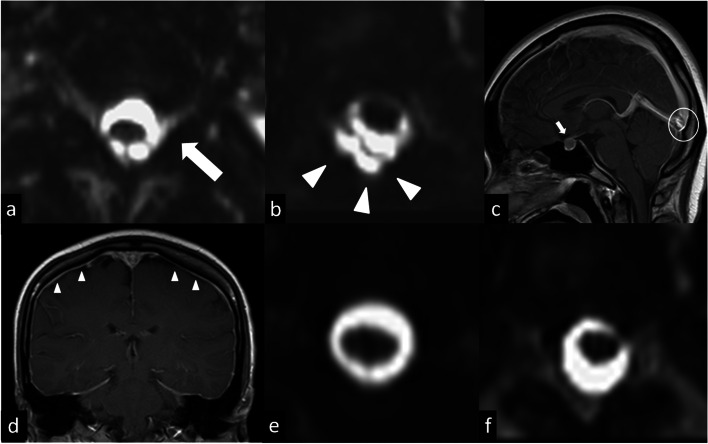
A 22 year-old woman suffered from orthstatic headache after spinal anesthesia. **a** Axial reconstruction MRM of C-T spine indicates spinal CSF leakage at left C5–6 neural sleeve (arrow). **b** Axial reconstruction MRM of C-T spine indicates abnormal posterior epidural fluid accumulation (arrowhead). **c** Gd-T1WI sagittal MR of brain indicates pituitary hyperemia (arrow) and dural sinus engorgement (circle). **d** Gd-T1WI coronal MR of brain indicates pachymeningeal enhancement (arrowhead). **e** and **f** Axial reconstruction MRM of C-T spine at corresponding levels to (**a** and **b**) 1 month later indicate no spinal CSF leakage and no abnormal epidural fluid accumulation

A 22-year-old woman was admitted for posterior mediastinal schwannoma surgery. Four days after surgery, the patient complained of an orthostatic headache with nausea and vomiting. Anesthesia-related spinal CSF leakage was suspected. The MRI and MRM images showed CSF leakage into the left C5-T1 neural sleeves (Fig. 3a) with posterior epidural fluid accumulation over C5-T3 (Fig. 3b). Additionally, conventional MRI demonstrated abnormal dural sinus engorgement (Fig. 3c), pituitary hyperemia (Fig. 3c), and pachymeningeal enhancement (Fig. 3d). The patient received intensive hydration and conservative bed rest treatment. The symptoms resolved, and a follow-up MRI performed 1 month later revealed no residual spinal CSF leakage or epidural fluid accumulation (Fig. 3e and f).

## Discussion

In this study, we determined that patients with SIH and those who have ≥9 spinal CSF leakages on MRM are at risk of hydration non-effectiveness. These patients also had longer hospitalizations.

The mean numbers of spinal CSF leakages for patients with PDPH and SIH in our study were 6.54 and 11.41, respectively. No statistically significant differences between these two groups and the number of leakages have been reported in the literature [18, 19]. Therefore, with the exclusion of disease severity, patients with spontaneous spinal CSF leakage are more prone to non-effective hydration than patients with PDPH.

According to the literature on post-lumbar puncture CSF leakage, not all patients are symptomatic or have clinically relevant symptoms [20]. Patients with PDPH had more periredicular leaks and segments of spinal epidural fluid accumulation than those without PDPH, and patients who ultimately required EBP had increased levels of epidural fluid accumulation, indicating that they have a higher disease severity [18]. In our study, only patients with PDPH and SIH were recruited. These patients may have a more severe disease phenotype than patients with asymptomatic spinal CSF leakage after dural puncture. Large needle size and younger age are reported risk factors for patients with PDPH who undergo dural puncture [18, 21, 22]. For the 11 patients with PDPH in our study, three patients required further EBP with a mean of 10 CSF leakages. The mean for the other eight patients was 5.25. Therefore, we determined that disease severity is a significant risk factor for treatment effectiveness.

In patients with SIH, EBP is commonly required for treatment [19]. Although the radiological parameters indicated no substantial difference between the hydration-effective and -noneffective groups, 46 patients required further EBP with a mean number of spinal CSF leakages of 12.17; the mean for the other 17 patients was 9.35. Therefore, ≥ 9 spinal CSF leakages may serve as an efficient clinical predictor for nonresponsive hydration, and EBP could be applied earlier.

Notably, the levels of epidural fluid accumulation were not considerably different between the two groups. In our study, nearly all patients had posterior epidural fluid accumulation. Patients usually remained in the supine position, and the extravasated CSF extended to the surrounding epidural space along the potential epidural space. Compared with the levels of abnormal epidural fluid accumulation, the number of spinal CSF leakages at neural sleeves may reflect disease severity more accurately. The supposition that anterior, compared with posterior, epidural fluid accumulation may more accurately reflect the severity of spinal CSF leakage has also been reported in the literature [14].

In terms of treatment efficacy assessment, this study evaluated the effectiveness of hydration using symptom improvement. Although symptom improvement is subjective, it is commonly used to clinically evaluate hydration efficacy [23, 24]. In contrast, radiological improvements are more accurate and objective. Moreover, it can further exclude delayed SDH, which easily occurs in patients with SIH [25]. However, during daily practice, we did not routinely assess the hydration efficacy using MRI in patients with spinal CSF leakage. Targeted EBP was administered without further imaging confirmation if intractable headache remained after hydration. Our data partially supported this intervention. In the current study, 25 patients responded perfectly to initial hydration, and none of these 25 patients experienced headache recurrence. Importantly, no residual radiological abnormalities were found in eight and nine patients who underwent MRI assessments within 1 and 6 months after hydration, respectively. However, scheduled imaging follow-up is recommended for patients with spinal CSF leakage after hydration in future studies.

Our study had several limitations. First, we used the treatment effect as the endpoint for patients receiving EBP. Patients may be sensitive to headaches and have different expectations regarding the treatment effect. Therefore, the clinical response and decision to receive further EBP may be subjective. Second, although PDPH is an independent factor predicting responsiveness to hydration treatment, further subgroup analysis focusing on PDPH patients was not performed due to the small sample size. The incidence of post-dural puncture CSF leakage is not as low as that of SIH, but patients with this condition are usually not referred to imaging studies due to good response to treatment or even spontaneous recovery. Studies with a prospective design that recruited larger sample sizes with scheduled imaging assessments may provide clearer findings.

## Conclusions

The number of spinal CSF leakages indicated on MRM is an efficient predictor of the effectiveness of hydration in patients with spinal CSF leakage. Patients with risk factors can receive EBP earlier to shorten the disease course and hospitalization.

## Data Availability

All data generated or analyzed during this study are included in this published article. The data that support the findings of this study are available from the corresponding author, upon reasonable request.

## Abbreviations

PDPH: Post-dural puncture headache
CSF: Cerebrospinal fluid
MRM: Magnetic resonance myelography
MRI: Magnetic resonance imaging
SDH: Subdural hematoma
EBP: Epidural blood patch
T1WI: T1-weighted images
ORs: Odds ratios
CIs: Confidence intervals
SIH: Spontaneous intracranial hypotension
